# Nonalpine Thyroid Angiosarcoma in a Patient with Hashimoto Thyroiditis

**DOI:** 10.1155/2013/901246

**Published:** 2013-11-18

**Authors:** Nadia Innaro, Elena Succurro, Giuseppe Tomaino, Franco Arturi

**Affiliations:** ^1^Department of Surgery, Policlinico “Mater Domini” of Catanzaro, Campus Universitario, Viale Europa, 88100 Catanzaro, Italy; ^2^Department of Medical and Surgical Sciences, University “Magna Graecia” of Catanzaro Policlinico “Mater Domini”, Campus Universitario, Viale Europa, 88100 Catanzaro, Italy

## Abstract

Thyroid angiosarcoma is an uncommon thyroid carcinoma and its incidence is the highest in the European Alpine regions. Thyroid angiosarcoma is also a very aggressive tumor that can rapidly spread to the cervical lymph nodes, lungs, and brain or can metastasize to the duodenum, small boewl, and large bowel. Although it is histologically well defined, clear-cut separation between the angiosarcoma and anaplastic thyroid carcinoma is difficult. A 49-year-old Caucasian female patient, born and resident in Southern Italy (Calabria), in an iodine-sufficient area, was admitted to the Surgery Department because she presented with a painless mass in the anterior region of neck enlarged rapidly in the last three months. After total thyroidectomy and right cervical lymphadenectomy, postoperative histological examination revealed the presence of a thyroid angiosarcoma with positive staining for CD31 and for both Factor VIII-related antigen and Vimentin and only partially positive for staining pancytokeratin and presence of metastasis in cervical, supraclavicular, mediastinal and paratracheal lymph nodes. The patient started adjuvant chemotherapy and she was treated for 6 cycles with Doxorubicin, Dacarbazine, Ifosfamide, and Mesna (MAID). After 22 months from surgery, the patient is still alive without both local and systemic recurrence of the disease.

## 1. Introduction

Thyroid angiosarcoma is a very rare disease. As previously reported by other authors, its incidence is the highest in the European Alpine regions and in some European areas as Northern Italy, Austria, and Switzerland it can constitute the 2–10% of malignant thyroid tumors [1–3]. In Southern Italy, no case of thyroid angiosarcoma has been described. Thyroid angiosarcoma is also a very aggressive tumor that can rapidly spread to the cervical lymph nodes, lungs, and brain or can metastasize to the duodenum, small bowel, and large bowel and induce severe bleeding [1, 4–6].

Although it is histologically defined as cleft-like anastomosing spaces lined by large, atypical cells of endothelial lineage [7], clear-cut separation between the angiosarcoma and anaplastic thyroid carcinoma is difficult because they yield nearly the same clinical prognosis and overlapping histologic findings. Indeed, the existence of thyroid angiosarcoma has been a matter of debate for many years because some authors believe that most if not all angiosarcoma are in fact “angiomatoid” anaplastic carcinomas [8–11].

Angiosarcoma treatment is complex and consisting of surgery, chemotherapy, and radiotherapy.

We report the first case of thyroid angiosarcoma identified in Southern Italy (Calabria), in nonalpine area.

## 2. Case Report

On November 2011, a 49-year-old Caucasian female patient, born and resident in Southern Italy (Calabria), in an iodine-sufficient area, was admitted to the Surgery Department because she presented with a painless mass in the anterior region of the neck. In her history, longstanding nodular goiter with regressive changes including intranodular hemorrhage was reported; the goiter had been noticed ten years ago and the patient was treated with levothyroxine. However, the neck mass enlarged rapidly in the last three months. Clinical examination of the thyroid gland showed a remarkable enlargement of the right lobe in which a nodular lesion was present. In contrast, the left lobe of the thyroid gland was nonpalpable. There was evidence of involvement of cervical lymph nodes.

Thyroid gland high-resolution ultrasonography confirmed the enlargement only of the right lobe that showed a morphology suggestive of an autoimmune thyroid disease and the presence of nodular lesion measuring 7.5 × 5.5 × 6 cm. In contrast, the left lobe was of normal size and normal structure. I^131^-scintiscanning showed that the nodule in the right lobe was hypofunctioning. The thyroid function was evaluated by measuring serum levels of free T_3_ (FT_3_), free T_4_ (FT_4_), and thyrotropin (TSH). Free T_3_ and free T_4_ levels were measured by RIA (Radim Spa, Roma, Italy). Plasma TSH levels were measured by IRMA (TSH Bridge, Biochem ImmunoSystems Italia, Spa, Milano, Italy). The results of thyroid function tests were within the normal range. The assay of the thyroid antibodies revealed a normal value of antithyroglobulin antibody and a positivity of the antithyroperoxidase antibody (8624 U/mL; normal value = 1–100).

The patient showed also iron deficiency anemia. Indeed, the blood values were the following: red blood cell count (RBC) is 4.17 × 10^6^/uL (normal value is 4.2–5.4 × 10^6^/uL), hemoglobin (Hb) is 8.8 gr/dL (normal value is 12–14 gr/dL), hematocrit 30.2 (normal value is 37–47%), mean corpuscular volume (MCV) is 72.4 fL (normal value is 81–99 fL), and mean corpuscular hemoglobin (MCH) is 26 pg (normal value is 27–31 PG), mean corpuscular hemoglobin concentration (MCHC) is 30 g/dL (normal value is 33–37 g/dL). Iron is 26 *μ*g/dL (normal value is 37–145 *μ*g/dL). White blood cell and platelet counts were within normal limits.

Neck computerized tomography confirmed the presence of a large vascular mass that measured 7.5 × 6 × 6 cm arising from the right thyroid lobe, which was described as necrotic and containing areas of calcification. There was significant mass effect with displacement of trachea to the left and there was compression of carotid bundle to the right. There was also involvement of both cervical and supraclavicular lymph nodes. There was no invasion of adjacent structures. Chest X-ray films showed clear lung fields.

After iron supplementation and normalization of the hemoglobin value, the patient underwent total thyroidectomy and right cervical lymphadenectomy. 

**Figure 1:**
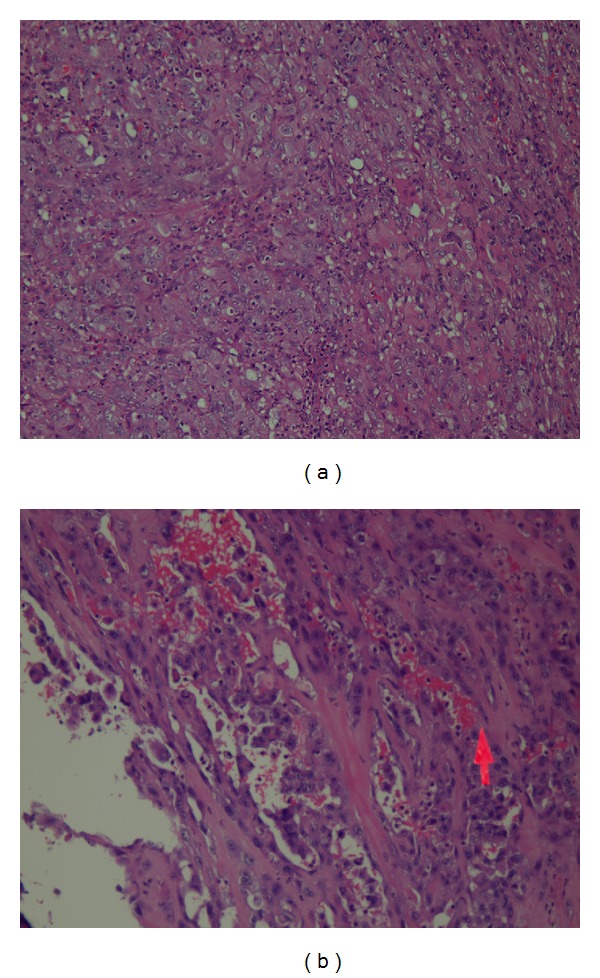
(a) Proliferation of cellular elements with epithelial appearance, with large vesicular nucleus, and with prominent nucleolus. (b) Areas consist of irregular spaces containing material blood bordered by atypical cells with intraluminal papillary projections.

Histological examination showed a well-circumscribed nodule confined within the thyroid and with large areas of necrotic fibrinous tissue. Microscopic examination disclosed a proliferation of epithelioid cells with enlarged pleomorphic nuclei and prominent nucleoli (Figure 1(a)). In several point of the tumor was possible to demonstrate the presence of irregular vascular areas containing hematic material bordered by atypical cells with intraluminal papillary projections (Figure 1(b)). The thyroid tissue unaffected by the tumor disclosed Hashimoto thyroiditis. Furthermore, in two cervical lymph nodes excised, the presence of metastatic tissue has been demonstrated.

**Figure 2:**
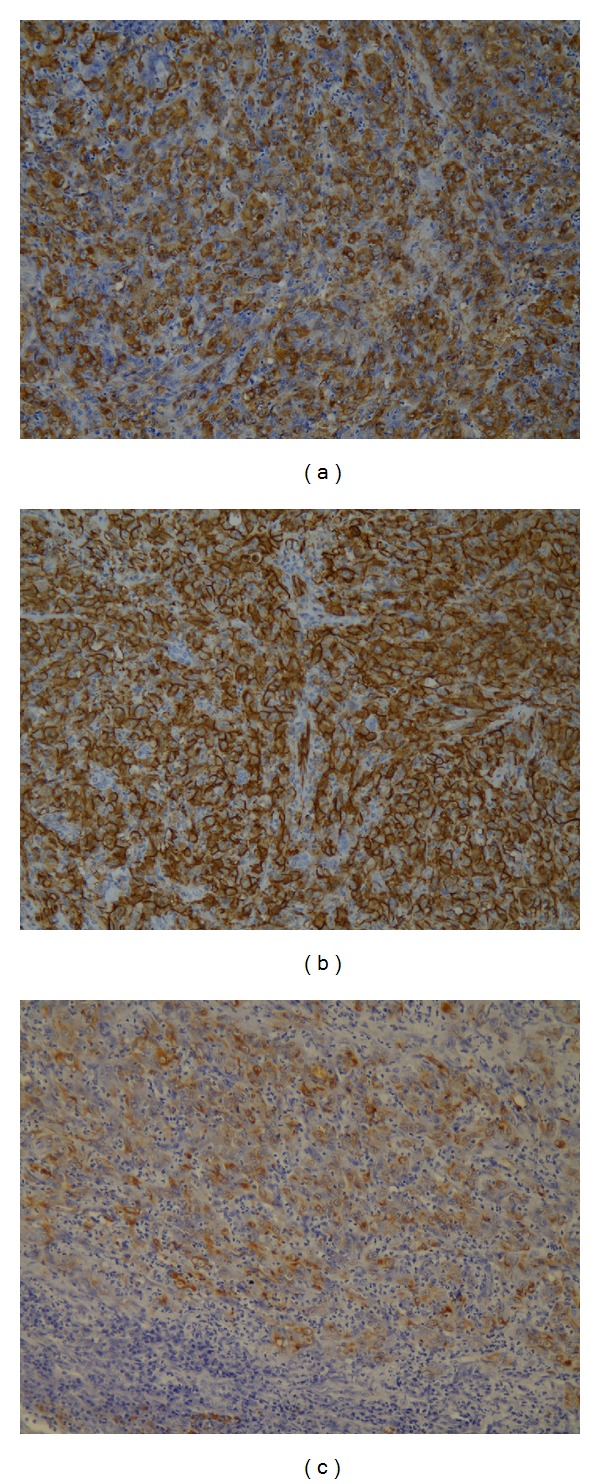
(a) Angiosarcoma cells show positivity for CD31 antigen (monoclonal mouse clone JC70A—Dako—40x). (b) Factor VIII-related antigen positivity in the angiosarcoma cells (policlonal rabbit—DAKO—40x). (c) Tumor cells express partially pancytokeratin (CK-) related antigen (cytokeratin monoclonal mouse clone AE1/AE3—DAKO—40x).

On immunohistochemical examination, tumor cells showed both extensive and intensive immunoreactivity for CD31 (Figure 2(a)); they were positive for Factor VIII-related antigen (Figure 2(b)) and Vimentin and only partially positive for pancytokeratin (Figure 2(c)). Tumor cells were negative for thyroglobulin, TTF-1, CD34, cytokeratin 7, cytokeratin 20, and cytokeratin 5/6.

Total body computerized tomography performed after surgical treatment revealed the presence of right cervical, supraclavicular, mediastinal, and paratracheal lymphadenopathy. There were no alterations like metastatic lesions in lung, liver, spleen, and/or other organs.

One month after the total thyroidectomy, the patient started adjuvant chemotherapy and she was treated for 6 cycles with Doxorubicin, Dacarbazine, Ifosfamide, and Mesna (MAID).

On June 2012, December 2012 and June 2013, after adjuvant chemotherapy, a total body TAC has been performed and there were no alterations like metastatic lesions in lymph nodes, lung, liver, spleen, and/or other organs. The patient is still alive without both local systemic recurrence of the disease after 22 months from surgery.

## 3. Discussion

In this paper we describe the first case of thyroid angiosarcoma identified in Southern Italy (Calabria), in nonalpine area. The incidence of this uncommon tumor is highest in European Alpine regions [1, 12–14], whereas few cases of thyroid angiosarcoma have been described in nonalpine areas. In the English literature have been reported only 22 cases of nonalpine thyroid angiosarcoma, including the case described in this paper [15–19]. In total, 11 of  22 patients (50%) with non-Alpine thyroid angiosarcoma were living in Italy. The explanation of this phenomenon is unknown. Thyroid angiosarcoma is more frequent in the female respect the male (female male ratio, 9 : 3) [16] and the age at diagnosis ranges from 50 to 88 years [16]. Even in our case the age at diagnosis was 49 years old and the patient showed both ultrasonographic and biochemical features of the Hashimoto thyroiditis. As previously reported [20], also our case was associated with long standing nodular goiter and regressive changes including intranodular hemorrhage. As suggested, these alterations could provide the anatomic substrate for the development of the vascular tumors [16]. Indeed, Sapino et al. [21] reported that prominent endothelial hyperplasia is present in goiter nodules that underwent intranodular hemorrhage, and reorganization phenomena of the Masson's hemangioma type and these events could represent a predisposing condition for subsequent neoplastic transformation [21]. However, there are considerable controversy about the true existence of thyroid angiosarcomas. Although it is histologically defined as cleft-like anastomosing spaces lined by large, atypical cells of endothelial lineage [7], some authors believe that most of the reported cases are anaplastic or undifferentiated carcinomas with angiomatoid features [3, 4]. Many authors believe that thyroid angiosarcoma is a variant of sarcomatoid carcinoma, in view of their nearly constant cytokeratin positivity in the neoplastic elements. On the other hand, other authors believe that true thyroid angiosarcoma of the thyroid does exist, though it is rarely observed [20]. Advances in immunohistochemistry have not helped to clarify this dispute.

Surgical resection to obtain the local control of thyroid angiosarcoma remains the still point of primary treatment. However, thyroid angiosarcoma is a very aggressive tumor. Indeed, it can rapidly spread to the cervical lymph nodes, lungs, and brain or can metastasize to the duodenum, small bowel and large bowel and induce severe bleeding [1, 4–6]. Metastatic disease is associated with poor prognosis and limits the mean survival time to a few months after diagnosis and surgical treatment. For this reason, the use of adjuvant chemotherapy and/or radiotherapy to obtain both the systemic control and local control of the disease has been introduced. Most patients develop postoperative early systemic metastasis, and it has been hypothesized that undetected micrometastases could be present at the time of the diagnosis [3]. Goh et al. [17], in a brief review on nonalpine epithelioid angiosarcoma, reported that the majority of cases with documented extrathyroidal extension at the time of the diagnosis had a uniformly poor prognosis with death occurring within 9 months. On the other hand, a good prognosis seems to be related to the absence of extraglandular extension at the time of the diagnosis as reported by Maiorana et al. [15]. In these cases, the patients are still alive with no evidence or residual disease also after 66 months after thyroidectomy (range of 21–66 months) [2, 15]. Although our patient showed a extraglandular tumour spread with involvement of cervical, supraclavicular paratracheal, and mediastinal lymph nodes at time of the surgical treatment, the adjuvant chemotherapy with Doxorubicin, Dacarbazine, Ifosfamide, and Mesna (MAID) was able to induce a reduction of metastatic disease. Indeed, the patient is still alive without both local and systemic recurrence of the disease after 22 months from surgery. 

In conclusion, thyroid angiosarcoma is an uncommon thyroid carcinoma and it is also a very aggressive tumor with a poor prognosis. Surgical resection remains the still point of primary treatment. However, our case showed that MAID combination chemotherapy may have an important role in the treatment of thyroid angiosarcoma in nonolder patients.
